# Addressing the Math-Practice Gap in Elementary School: Are Tablets a Feasible Tool for Informal Math Practice?

**DOI:** 10.3389/fpsyg.2017.00179

**Published:** 2017-02-21

**Authors:** Sara T. Stacy, Macey Cartwright, Zjanya Arwood, James P. Canfield, Heidi Kloos

**Affiliations:** ^1^Department of Psychology, Michigan State UniversityEast Lansing, MI, USA; ^2^Children’s Cognitive Research Lab, Department of Psychology, University of CincinnatiCincinnati, OH, USA; ^3^School of Social Work, University of CincinnatiCincinnati, OH, USA

**Keywords:** IXL, technology, math learning, ipad, math education

## Abstract

Students rarely practice math outside of school requirements, which we refer to as the “math-practice gap”. This gap might be the reason why students struggle with math, making it urgent to develop means by which to address it. In the current paper, we propose that math apps offer a viable solution to the math-practice gap: Online apps can provide access to a large number of problems, tied to immediate feedback, and delivered in an engaging way. To substantiate this conversation, we looked at whether tablets are sufficiently engaging to motivate children’s informal math practice. Our approach was to partner with education agencies via a *community-based participatory research* design. The three participating education agencies serve elementary-school students from low-SES communities, allowing us to look at tablet use by children who are unlikely to have extensive access to online math enrichment programs. At the same time, the agencies differed in several structural details, including whether our intervention took place during school time, after school, or during the summer. This allowed us to shed light on tablet feasibility under different organizational constraints. Our findings show that tablet-based math practice is engaging for young children, independent of the setting, the student’s age, or the math concept that was tackled. At the same time, we found that student engagement was a function of the presence of caring adults to facilitate their online math practice.

## Introduction

“Math is hard.”

Will, 11

To what extent does math competence depend on informal math practice (IMP)? Surprisingly, there is very little research on this question, which stands in sharp contrast to the amount of research on informal reading practice. We argue that IMP faces practical barriers: Math practice is far more difficult to carry out informally than reading practice, creating a “math-practice gap”. Thus, to help students develop math competence, a solution to the math-practice gap needs to be found. In the current paper, we look at the use of online math apps as a possible solution. Specifically, we ask whether tablets are sufficiently motivating for children to engage in math practice outside of school-required assignments and homework.

In what follows, we will first justify the need for IMP to supplement in-class math education, focusing specifically on elementary-school arithmetic. We then discuss the practical barriers to IMP and how tablets could address these challenges. Central to our argument is that math practice needs to be interactive and individualized, providing students with a sustained positive experience of success. This cannot be done easily without online support, which is where research on tablet feasibility comes in. We carried out such a feasibility study, using a community-based participatory research design. While this method does not allow for a precise control of variables, it has the advantage of maximizing ecological validity.

### The Nature of Math: How Important is Practice?

The importance of practice is well known: No matter what the skill, practice is likely to benefit competence (e.g., Kanive et al., 2014). At the same time, mindless drill has fallen out of favor, along with memorization and busy-work (cf., Delpit, 2012) Indeed, a search through the literature reveals a focus on didactics (how to convey a math concept) – more so than a focus on math practice. This leaves little empirical guidance to determine what kind of math practice might be best. We will go another route to look at this question: We will first examine the mental activities needed to solve a math problem. We will then contrast them with the mental activities that are needed to read. Reading, as it turns out, is a domain that has enjoyed a long track record of established findings on informal practice (e.g., Rasinski, 1990; Pikulski and Chard, 2005). Thus, a side-by-side comparison between math-related mental activities and reading-related mental activities allows us to make inferences about math practice.

Our focus is specifically on elementary-school arithmetic and the concepts outlined in the Common Core State Standards Initiative (2011). They include operations with integers (i.e., addition, subtraction, multiplication, division), operations with fractions (e.g., ordering of fractions on the number line, equivalent fraction, improper fractions) and operations with decimal numbers (e.g., place values, correspondence between decimals and fractions). Overall, this domain has several advantages for the purposes of the current feasibility study: For example, there is a high variability in concepts (e.g., National Council of Teachers of Mathematics, 2000), making it possible to derive generalizable claims. Elementary school is also the time during which children learn to read, giving credence to a side-by-side comparison. **Table 1** summarizes our reflections on mental activities likely to be required at each grade level. As can be seen from the table, the challenges for the mind are likely to be far greater for math than for reading, independent of what is covered at each grade level. In the remainder of this section, we fully describe these differences.

**Table 1:** Assumed mental activity for reading and math in K-6 grades.

Grade/Subject	Content	Challenge for the Mind
**Kindergarten**
Reading	Letter system	Attention to detail
Math	Number system	Attention to detail, precision, abstractness
**1st Grade**
Reading	Reading words	Attention to detail, fluency
Math	Addition/subtraction	Attention to detail, precision, fluency
**2nd Grade**
Reading	Reading sentences	Attention to detail, fluency
Math	Multi-digit operations	Attention to detail, precision, fluency, relational reasoning, alternate meanings
**3rd Grade**
Reading	Reading paragraphs	Fluency
Math	Multiplication/division	Abstractness, precision, interfering fluency
**4th Grade**
Reading	Reading essays	Fluency
Math	Fractions	Abstractness, attention to detail, precision, interfering Fluency, alternate meaning, relational reasoning
**5th Grade**
Reading	Reading chapters	Fluency
Math	Decimals	Abstractness, attention to detail, alternate meaning
**6th Grade**
Reading	Reading chapter books	Fluency
Math	Negative integers	Interfering fluency, attention to detail, alternate meaning

In Kindergarten, math is primarily about mapping symbols to quantities, which requires attention to detail. This mimics the mental activity that is required for reading. But beyond attention to detail, the mind also needs to apply a precise counting routine. And it needs to detect the abstractness of number (i.e., that a number refers not just to entities, but also to time, distance, or events). None of these mental activities are required for reading, suggesting that the amount of practice needed for math may already be higher than the amount of practice needed for reading.

In 1st grade, math is about addition and subtraction, which is yet another set of routines. By 2nd grade, children need to expand this fluency to multi-digit numbers, which further adds to the set of precise routines. Note that multi-digit numbers provide mental challenges of their own: Consider, for example, the numbers [*20*] and [*02*]. Even though the individual digits are the same in both cases, their meaning is vastly different, even unconventional in the latter case. Thus, the meaning of a digit is defined by its spatial location – a feature that has very little ecological validity for children (i.e., few entities change meaning because of where they are in relation to other entities). Furthermore, there is no statistical regularity or context that children could rely on to derive meaning. The mind must provide meaning entirely on its own.

Notice, from **Table 1**, that the complexity of reading has reached its peak by the end of 2nd grade. After this grade, it is simply a matter of becoming a fluent reader. In contrast, conceptual challenges for math keep piling on. For example, in 3rd grade, a whole new domain is introduced: multiplication and division. Unlike addition and subtraction, these operations are not grounded in everyday language, thus requiring a certain level of abstractness. Furthermore, these operations come with a set of procedures and routines that need to be followed precisely. The mind also needs to attain a certain fluency in these procedures – one that interferes with the fluency acquired for addition and subtraction. Finally, the fluency in multiplication and division cannot be achieved through the gradual removing of a scaffold, but requires studious memorization – all enormous challenges for the mind (e.g., Welsh et al., 1991; Zelazo and Müller, 2002).

Then comes 4th grade – and with it a whole slew of conceptual challenges of abstraction, precision, and fluency. In this grade, children need to master fractions, which requires nothing less but to re-learn the very meaning of a number. Prior to fractions, numbers referred to whole quantities. Now numbers refer to either the number of parts (numerator) or the total number of parts (denominator). Both meanings must be accessible smoothly, and they must be understood in relation to each other. The challenge continues with decimal numbers and negative numbers (5th and 6th grade): Numeric symbols change in meaning because of a miniscule detail (e.g., [*2.0*] vs. [*0.2*]; [*2-*] vs. [-*2*]). The location of something as little as a decimal point, or of something as little as a negative sign, decide on the meaning of a number.

Consider, by contrast, what it takes to make sense of printed material. Individual letters appear in stable configurations that have largely unique meanings. For example, the word [*duck*] largely means [*duck*], no matter what context it appears in. When a word has more than one meaning, as is the case for homophones or metaphoric expressions, a readily available context will disambiguate the meaning. Rather than having something as miniscule as a dot to provide meaning, the entire sentence is available to give clues. There are ambiguities, of course (e.g., in [*The old man the boat*], [*man*] is used unexpectedly as a verb and [*old*] is used unexpectedly as a noun). But these ambiguities are exceedingly rare, and the larger context of the story often provides the necessary clues to generate meaning.

Taken together, we have shown that the nature of math is likely to be very challenging for the mind, namely from the very beginning, and exceedingly more so with every new grade. This is attributed to the need for fluency; the need for abstraction that changes with the context; the need for attention to detail, miniscule as the detail might be; the need to keep in mind different meanings and switch between them fluidly; and the need for relational reasoning. This analysis of math content (vis-à-vis reading content) should make it abundantly clear that math competence depends crucially on practice, more so than reading competence. It is even possible that a lack of sufficient math practice could conceal the source of a math learning difficulty. Thus, the shortage of research in this area is likely to be a problem for the field of math education. It is urgent to investigate math practice and how it can be done most effectively. In the next section, we turn to this question, focusing specifically on the barriers to math practice and how they can be overcome.

### Math Practice: What Does It Take?

What kind of practice is most beneficial? The American Academy for Pediatrics (AAP) encourages parents to read with their children long before children reach the age of formal schooling (American Academy of Pediatrics News [AAP], 2014). Once school starts, there are multiple ways in which children are encouraged to practice, for example, through library memberships. Indeed, the 2013 report of the Pew Research Center found that 70% of interviewed parents visited a public library with their child in the past 12 months. Furthermore, 55% of children owned their own library card, and 87% of children’s visits to the library ended in children borrowing a book. Even without family support, many schools have their own libraries to provide children with exposure to reading materials and make reading practice attainable. Formalizing these efforts, many schools have adopted the Accelerated Reader program to further encourage and track reading practice (Stefl-Mabry, 2005).

Furthermore, the AAP (2014) recommends for parents to establish a daily reading routine and allow children to choose the books themselves. Along the same lines, the Accelerated Reader program encourages children to choose their own books and work toward personalized reading goals (Renaissance Learning, 2016). The idea is that individualized practice, carried out frequently and in the context of a positive experience, is likely to strengthen reading competence (e.g., Nunnery et al., 2006). This approach agrees with the theoretical models of learning motivation, namely to provide children with mastery, autonomy, and purpose (Pink, 2011). Reading at one’s own skill level allows students to feel competent; being able to choose the reading material allows for a high degree of autonomy; and the joy that is part of reading provides purpose to the activity.

A very different picture emerges with math practice. There is no general call for students to practice math at their own level. Instead, math practice is largely confined to school assignments and prescribed homework. The content and pace of such formal practice is dictated by the curriculum, leaving students little choice. For example, students are expected to complete all math problems on their worksheet or homework, by a deadline, and they are judged on their performance. The consequences of this state of affairs is much worse for students who are already behind in math. Having to work on something that is above their competence level is likely to lead to a negative experience and rob students of a sense of purpose and mastery (e.g., Slavin and Lake, 2008; Re et al., 2014; Kucian and von Aster, 2015).

An alternative is to encourage students to practice math at home, mimicking the initiatives for self-guided reading, over and above homework. However, this is likely to face substantial practical barriers: It is rather difficult to orchestrate self-guided practice and encourage children to carry it out. An adult would need to develop practice problems that have the appropriate difficulty level for the child. The adult would also need to provide meaningful feedback to the child, to allow for discovery of potential gaps in the required skills. On top of that, the adult would have to provide a positive context and motivate the child to practice math. Together, this provides a substantial time investment and competence of an adult.

Apps on touch-screen tablets might be a viable solution: practice problems are already pre-determined, they are delivered in a playful format, and they provide instant feedback – all without the time investment of a trained adult (e.g., Kyanka-Maggart, 2013; Warman, 2014; Hilton, 2016). For instance, Kucian et al. (2011) found that 8- to 10-year-olds, instructed to practice math at home for 15 min a day, 5 days a week for 5 weeks, showed improved performance compared to pre-test performance. The likely benefit of computer-assisted interventions to support math competence has made it become more embedded in the educational context (e.g., Fuchs et al., 2006; Räsänen et al., 2009; Burns et al., 2010; Kesler et al., 2011; Kucian et al., 2011; Stickney et al., 2012; Doabler and Fien, 2013; Gross and Duhon, 2013; Jansen et al., 2013; Kanive et al., 2014). Here, we seek to expand these efforts and look at whether online apps are conducive to IMP.

We chose the math app IXL.com, without necessarily endorsing it over and above any other practice programs (see commonsensemedia.org, for other math practice apps). The IXL app currently has approximately 5.6 million school licenses and 400,000 family licenses in use (IXL staff, personal communication, October, 2016). It provides extensive opportunity to practice math skills relevant to the Common Core, ranging from pre-K basics to high school pre-algebra, algebra, and pre-calculus. This continuity in math skills makes it possible to find the appropriate difficulty level for a child, independent of grade level, background information, or motivation. Math practice problems are organized by grade, math topic, and problem sets. And each problem set features an example problem to facilitate the decision about what to practice. The setup delivers encouraging feedback when a math problem is solved correctly, it uses a point system that advances like a video game, and it downplays mistakes. When children make a mistake, the app provides a brief explanation of the concept, allowing children to learn from their mistakes, if they so choose.

### Overview of Our Study: A Community-Based Participatory Research Approach

Our specific approach followed the design of community-based participatory research (CBPR). This approach emphasizes that research activities are decided upon in partnership with community agencies, namely to meet the needs of the community and maximize the likelihood that the activities benefit their members (e.g., Minkler and Wallerstein, 2003). Even though CBPR is rare in the context of math learning, it offers unique strengths to feasibility studies. CBPR allows the research to consider real-life complexities, including the presence of multiple stakeholders, as well as their unique constraints, priorities, and challenges. Such complexities often pose substantial hurdles for experimental results to be translated into a viable program and implemented on the ground – even very promising experimental results. CBPR makes it possible to anticipate these hurdles and help find ways to circumvent them.

At the same time, CBPR is not without shortcomings. Most importantly, the details of the methods are not entirely up to the researchers. They are instead designed in collaboration with the community partners, considering the existing structures within the organization and the goals of the community. Consequently, the research activities at a site are unique, mapped onto the needs of the community and the realities on the ground, with far less regard for precise data collection, control groups, and randomization. To circumvent these shortcomings and obtain meaningful results, our strategy was to implement the same general intervention in more than one setting.

For the current purposes, we partnered with three organizations, all of them serving elementary-school children from low-SES communities (two elementary schools and one non-profit organization). The effect of SES on early math achievement has been explored widely (e.g., Griffin et al., 1994; Jordan et al., 2002; Tucker-Drob and Harden, 2012). Children from low-SES communities are unlikely to have broad and frequent access to touch-screen tablets (cf., Bradley et al., 2001; Galindo and Sonnenschein, 2015). This allowed us to establish math-practice feasibility for a population that might lack extensive familiarity with this medium.

Working together with community partners, four settings were used to introduce tablet-based math practice. The first setting was a weekly enrichment program with one-on-one mentoring. Our program took place during one of those enrichment events, to observe tablet feasibility in a large group of child–adult pairs. The second setting was a summer camp implemented with camp counselors and volunteers. Our program took place for approximately 40 min per week, for five sessions, the goal being to observe large-group feasibility when one-on-one pairing between children and adults was not possible. The third setting was an in-school tutoring program. Here, we integrated the tablet-based practice with ongoing paper-and-pencil practice, to understand how the tablet-based practice interfaces with traditional tutoring. Finally, the fourth setting was an after-school program, offered alongside after-school homework help. Here, our program was carried out exclusively with tablet practice, to explore voluntary attendance to a math-practice program.

Our general approach was to bring touch-screen tablets to each of the settings and to observe the behavior of children as they engaged in math practice. While adult volunteers were always present, whether for small group or one-on-one support, their role differed slightly from that of a tutor (cf., Fuchs et al., 2008, 2013). Volunteers were asked to merely encourage children, not actually provide didactic support. This was done to get a better sense of a child’s spontaneous interaction with the tablets. Note that we did not look at the effect of tablet use on math competence, as this was not possible in the current study design. Nevertheless, our design provides an important window into the question of whether tablets with math apps are a feasible tool for math practice.

## Materials and Methods

**Table 2** provides an overview of the settings used for our observational study, including the ways in which they differed as a result of our CBPR design. For each setting, iPad tablets were used and outfitted with the IXL math practice app. We used a bulk of 30 generic log-ins that were shared between children across the different settings. Adult facilitators were available to guide children’s math practice and provide encouragement throughout the sessions. In what follows, we describe each setting, the students, and the math practice activities that were carried out at each setting.

**Table 2:** Overview of settings.

	(1) Enrichment Program	(2) Summer Program	(3) In-School Program	(4) After-School Program
Students	*N* ≅ 30	*N* = 111	*N* = 31	*N* = 19
Age range	9–11	6–11	9–11	9–12
Duration	1 h	4 h	10 h	20 h
Attendance	Mandatory	Mandatory	Mandatory	Voluntary
Type of facilitation	One-on-one	Small group	One-on-one	One-on-one
Adults training	Minimal	Medium	Minimal	High

### Setting 1: Enrichment Program

#### Cohort

Students were 3rd, 4th, and 5th graders at risk of, or currently experiencing transient living situations (determined by the school). They participated in a weekly enrichment program, organized by a local nonprofit agency that serves youth experiencing homelessness. Its goal was to provide students with unique experiences throughout the course of the school year, namely by pairing them up with a college mentor during each meeting. Each enrichment event occurred weekly for 90 min, and our intervention took place during one of those enrichment events.

#### Math-Practice Intervention

The 90-min math-practice intervention was presented as ‘Math Olympics’, complete with team flags, score charts, and medals. There were four ‘competitions’ students were asked to participate in, namely addition, subtraction, multiplication, and division, the goal being to complete as many problems as possible within a certain amount of time. Student–mentor pairs were organized into teams, although each student–mentor pair worked on their own math problems on the tablet. Mentors were instructed to help students find a problem set that they could complete independently: not too easy and not too difficult. Then students were given a few minutes to practice, which allowed mentors to check whether their choice of problem was appropriately challenging. Once mentors were confident that they had chosen a good problem set for the students, the competition started. At the end of the competition, the team that won the most games received a prize and the other teams received smaller prizes for participation.

### Setting 2: Summer Program

#### Cohort

Students were in grades K-6, ranging in ages between 6 to 11 at the onset of the program. The students were selected to be a part of a 7-week summer camp because they were at risk of, or currently experiencing homelessness (as determined by the program administrators). They were recruited from personnel within local homeless shelters and case managers of local schools. There was no charge to attend the summer program, and general attendance rate was about 70%. Students were organized into three groups of 20 to 25 students per classroom, based on their age. Each group had a teacher, an instructional assistant, and a college mentor to lead the group, in addition to a small group of volunteers who supported the program (3 to 5 per classroom).

#### Math-Practice Intervention

Our intervention took place once a week, for a total of five sessions of approximately 40 min per group. At the beginning of a session, students were given a tablet and told to start with a common problem set. This initial problem set was chosen in such a way that all children in a classroom could complete it, as per camp counselors and prior sessions. Once children completed the common ‘warm-up’, they were asked to find a problem set that was appropriately challenging for their level, with the help of adult facilitators. Overall, only minimal training was given to the facilitators; they were merely instructed to assist the students in finding problems that were tailored to their ability and to motivate the students during the session. Due to the high number of students (compared to the number of adults), student-adult pairing was not possible. Thus, students were typically in groups of five, with one adult per group. Sometimes, parents joined in as well, working one-on-one with their children.

### Setting 3: In-School Program

#### Cohort

Students were 4th graders ranging in age from 9 to 11 at the onset of the program. All students attended an inner-city public school that serves families from disadvantaged communities: According to this school’s most recent Ohio School Report Cards (2016), 99% of the students are considered economically disadvantaged, 97% of them are African-American, and only 14% of 4th-graders passed the state test in math. The setting was a tutor program held once a week for 45 min during school hours for students with low performance in math (per teacher recommendation). Each student was paired up with a college mentor to work with. For each meeting, a work-sheet was provided and mentors were asked to help their student the way they see fit.

#### Math-Practice Intervention

Our intervention took place during the tutoring program. In addition to the work-sheets, college mentors were also given tablets with the math app. Students were asked to use the tablets to practice single-digit multiplication facts at the beginning of each session. The college mentors were also asked to find appropriate problem sets for the student. Specifically, they were told to work on worksheets administered by the school staff and switch to the tablet practice when the worksheet problems were either too difficult (i.e., they perceived the students to benefit from extra practice) or too easy (i.e., they perceived the student to benefit from more challenging problems).

### Setting 4: After-School Program

#### Cohort

Students were in grades 4 to 7, ranging in age from 9 to 12 at the onset of the program. All students at this location attended an urban private school, where 85% of the students qualify for free or reduced lunch and the large majority of students are African American. The students were recruited to participate in this intervention due to a need for additional help with math (as determined by their math teacher). Many of these students attended an already existing after-school tutoring program.

#### Math-Practice Intervention

Our intervention took place alongside the existing tutoring program, offered on different days so as to not interfere with the ongoing homework help. Our intervention was offered twice a week during a 7-month period, and students had the freedom of choosing when to attend (once or twice a week). The students were paired one-on-one with a facilitator to practice. Facilitators were encouraged to assist students in finding problems tailored to their ability and to motivate the students during the session. Students received incentives for attendance, which included snacks during each session, as well as larger incentives when they reached attendance milestones. Prior to the onset of the program, facilitators participated in a 3-h training session focused on protecting children from harm; and they participated in a 2-h training session designed to help them interact with children.

### Measures

Given the nature of this community-based participatory research project, settings differed in what kind of data could be collected to evaluate feasibility of the math-practice intervention (see **Table 3** for an overview). Use of data was approved by the institutional review board, following ethical guidelines for research. In what follows, we describe each of the measures and how they were analyzed, after which we turn to describing our findings, separately by setting.

**Table 3:** Data collected, separated by setting.

	(1) Enrichment Program	(2) Summer Program	(3) In-School Program	(4) After-School Program
Informal observations	Yes	Yes	Yes	Yes
Systematic observations	No	Yes^1^	Yes^2^	Yes^2^
Math attitude survey	No	No	Yes	Yes
Math competence (T_5_ and T_10_ of WJ IV)	No	Yes	Yes	Yes
Student exit survey	No	No	No	Yes
Facilitator exit survey	No	No	Yes	No
Teacher interview	No	Yes	No	No

*Systematic observations differed in whether the 5-point Likert scale measured (1) the degree of happiness or (2) degree of nervousness. Math fluency and calculation competence was determined using standardized subtests from the Woodcock–Johnson IV battery*.

#### Informal Observations (Used in All Settings)

Informal observations are an important part of community-based participatory research, making it possible to describe the impact of an intervention in ecologically valid ways (e.g., Malterud, 2001). Observations were carried out by the authors, all of whom have been trained in the best practices of observational research (e.g., on how to minimize reflexivity and preconceptions, and how to maximize transferability). Field notes served as basis for the qualitative analyses.

#### Systematic Observations (Used in Settings 2–4)

During each session, facilitators were asked to record the problem sets that a child worked on. Facilitators also recorded how the child felt after each session (“How do you feel about doing math today?”). A 5-point Likert scale was used, each level being conveyed with a line drawing of a face (e.g., happy face, sad face). We used two versions of this scale, one version assessing degree of happiness (ranging from feeling ‘very sad’ to ‘very happy’), and another version assessing the degree of nervousness (ranging from feeling very nervous to not nervous at all). Each child was presented with only one type of scale. Results were analyzed in terms of the number of sessions children participated in the type of problems children worked on, and their rating of the sessions.

#### Math Attitude Survey (Used in Settings 3 and 4)

We developed a survey to assess children’s attitudes toward math at the onset of our program. It included items on how they feel when they are asked to complete math problems, whether they picture themselves in a job that will involve a lot of math, and how they feel about their math skills (compared to girls, boys, or others in general). We also asked them to report on their coping mechanisms when faced with a challenging math homework. In a series of yes–no items, five items were specifically geared toward coping behaviors that are productive (e.g., getting motivated; “Do you ask somebody for help?”), and five items were specifically geared toward coping behaviors that are negative (e.g., getting distracted; “Do you try to get out of having to do it?”) The difference between the number of positive versus negative coping behaviors reflects the degree of successful coping strategies a student had (ranging from -5 to +5). Results were analyzed in terms of average responses across items. Regarding validity and reliability, this measure is still under psychometric testing. For this reason, we treated each item individually, as single-item indicators, directly expressing the desired construct regarding a given attitude. Rather than report the findings as a math attitude “score,” we merely counted the number of positive and negative items.

#### Math Fluency and Calculation Competence (Used in Settings 2–4)

To get a better sense of children’s math skills, we measured math fluency and calculation competence, using two subscales from the Woodcock-Johnson test battery (Version IV). The subscale T_10_ measures math fluency with a 3-min-long timed test. It consists of two pages of simple operations with one-digit numbers, including addition, subtraction, and multiplication. The subscale T_5_ measures a student’s calculation competence. Students are instructed to do as many problems as they can until it gets too difficult, with no time limit. Items on this test range from simple operations (e.g., single digit addition, subtraction, etc.), to more difficult problems (e.g., multi-digit division, fractions, operations with negative integers, etc.) to advanced problems – too advanced for our purposes (e.g., logarithmic operations, calculus operations, etc.) Both subscales return the child’s grade equivalent score. Results were analyzed in terms of average grade equivalence at the onset of the program (math fluency; calculation competence), as well as in terms of amount of change in these measures, from the beginning of the program to the end.

#### Student Exit Survey (Used in Setting 4)

We developed a survey to assess student perceptions of the program after it was completed. This was a standard satisfaction-type survey that directly probed expressed constructs. Our reporting of findings mirrors this, by simply reporting counts, and not a composite score for the exit survey. Students were told that their answers will be used only to gather information about the program and would not impact their grades or be shared with teachers or parents. The first part of the survey used open-ended questions about likes and dislikes of the program (e.g., “What did you like about the program?”). The second part had a series of items that measured children’s beliefs about the program on a 3-point Likert scale. For example, children were asked to judge how much the program helped them with math (with the answer options being: “not very much”, “a little bit”, and “a lot”). The survey was one page long and took about 5 min to complete individually. Results were analyzed qualitatively, to get at children’s experience of the program.

#### Facilitator Exit Survey (Used in Setting 3)

We developed a paper-and-pencil survey for facilitators, administered at the end of the intervention. This too was a satisfaction-styled survey with single items directly expressing given constructs. Facilitators were asked to rate the frequency with which they used the math practice app (compared to the paper-and-pencil worksheets used in this setting), and to describe the most common ways in which they used the app. Facilitators were also asked to describe the strengths and weaknesses of the math practice app and tablet use, and to provide suggestions for intervention improvement. Results were analyzed qualitatively, to shed light on the experience of facilitators.

#### Teacher Interview (Used in Setting 2)

We developed a semi-structured interview for teachers, administered at the end of the program, to examine teachers’ thoughts and feelings about our tablet-based math intervention. The interview had three questions, but teachers were allowed to express new ideas and concepts outside of the line of questioning. First, teachers were asked “What are your thoughts on the math program?” Next, they were asked, “What works about the math program?” Finally, teachers were asked, “What would you change about the math program?” Each interview took approximately 10 min to complete. Field notes were used to record comments and were analyzed for themes. Results were analyzed qualitatively, to shed light on teacher experience.

## Results

### Setting 1: Enrichment Program

#### Informal Observations

Results for this setting pertain merely to our informal observations, but they are nevertheless telling. Overall, students involved in this setting were visibly engaged in the math practice from start to finish. There were no behavioral problems, which is unusual for a size of about 30 students working on math. Additionally, students were able to use the tablets and the math practice app with minimal instruction, pointing to the user-friendly design. At the end of the session, the organizers of the enrichment program commented on the positive behavior and engagement of the students while practicing math. One of the organizers even stated that you could hear ‘the drop of a needle’ because the students were so focused during the session. Thus, this setting provided the first indication that tablet-based math practice has the potential to engage young children and motivate them to practice math. Given this success, the organizers of the enrichment event invited our team to implement our intervention in their summer program (Setting 2).

### Setting 2: Summer Program

#### Informal Observations

Students were often quite excited when we arrived to their classroom and tablets were handed out. Many worked silently and diligently during the practice, showing no difficulty with using the tablet and the app. Despite the little amount of supervision and instruction, the students could navigate the app and tablet, and they worked independently throughout the entirety of the session. At the same time, there were some challenges, most notably since there were far more children than facilitators. Some of the older students were bored with the problem sets that were chosen at the onset of a session, while younger students were overwhelmed with the chosen problem set. Students had difficulty finding problems that are appropriately challenging, and even facilitators sometimes struggled with what to practice next.

#### Systematic Observations

Students attended between one to five sessions, with the average attendance rate being 2.75. Students in Grades 1 and 2 typically worked on counting and picture-based addition problems. Students in Grades 3 and 4 typically worked on addition, subtraction, and multiplication. And students in Grades 5 and 6 typically worked on multiplication and fraction problems. Students in all grades usually reported that the sessions they participated in were either fun or super fun, with only 14 students ever reporting that the session was either ‘bad’ or ‘super bad’ (which is less than 5% of the responses). This high level of reported enjoyment was confirmed by our observations.

#### Math Fluency and Calculation Competence

Students performed largely at grade level when entering the summer program. First-grade fluency was even above grade level. However, students 3rd grade and older often performed below grade level, especially for math fluency (being about one grade level behind). While calculation competence for older grades was typically at grade level (on average), there was very high variability in individual student scores, far higher than was observed for the younger students. Older children therefore are more strongly in need of a math intervention. Across the summer program, almost all the younger children improved in math fluency (82%). However, only approximately half of the older children did so (52%). In terms of calculation competence, only the first-graders improved as a group (by half a grade level on average). All other averages were lower at the end of the program, compared to at the onset. While these findings cannot be attributed to our intervention (positive or negative), they are nevertheless informative in terms of the challenge that comes with what a successful program needs to accomplish to counter the summer learning loss (cf., Cooper et al., 1996).

#### Teacher Interview

Teacher responses were in line with our observations. They noted the benefits of tablet learning, even for children who were known to have behavioral problems or math learning difficulties. Given that children differed significantly in their math ability in this setting, teachers expressed the importance of children working at their own skill level and at their own pace, without being pressured to perform at the level of other students in the class. Teachers also mentioned structural issues that provided a challenge to the tablet intervention, including the Wi-Fi connectivity. Even so, all teachers advocated for the tablet intervention to return in the next summer due to the reportedly outstanding results they felt it had on the students.

### Setting 3: In-School Program

#### Informal Observations

Students were visibly eager to begin their session with single-digit multiplication practice using the math app on the tablets. However, multiplication was a challenge for some of the students, and those weakest in math would sometimes get frustrated. In these instances, the facilitator would intervene and move them to something simpler, often single-digit addition. Students were often reluctant to put the tablets away when it was time to work on the pencil-and-paper worksheets, and they would frequently ask to switch back to the tablet. Especially when the worksheet was too easy or too difficult, students often went back to the tablet, working on problems that were either more challenging or simpler than the worksheet. In one instance, a child completed a worksheet on calculating rectangular area and perimeter within a few minutes. Rather than continue to work on material that was not challenging or engaging, the facilitator found a problem set on the tablet for calculating area and perimeter of more complicated shapes.

The tablet was also used to target specific weaknesses that were leading to further problems. For example, when difficulty in rounding decimals was traced to a lack of understanding place values, one student was directed to a problem set that focused specifically on identifying place values. The student had been struggling with rounding decimals for numerous weeks, but it took only one session of math-app practice to master this skill. Overall, students were observed to benefit from the tablets in ways that would have been difficult to address with class-wide paper-and-pencil practice.

#### Systematic Observations

Students used the tablets for an average of 5.56 sessions. The most frequently reported type of tablet practice was multiplication (75%), followed by fractions (24%). When asked how students felt about a session, a large majority of children (81%) reported ‘not nervous’ on all of the sessions.

#### Math Attitude Survey

All students reported liking at least some part of math. However, almost half of them reported disliking some part of math (43%), and over a third of them reported to be at least somewhat nervous when having to do math (36%). Many children hoped to get a job that involves a lot of math (75%), and they consider themselves to be good or very good at math (75%). Interestingly, over half of the children believed they are worse than girls in general (55%), compared to being worse than boys in general (28%). In other words, for this group of children, girls were more likely to be perceived as math competent than boys. In terms of coping strategies, the average degree of successful coping was 2.82, with only three children obtaining a score of 0 or below (i.e., reporting no more motivating than distracting behaviors). One child obtained a score of 1 (i.e., reporting 5 motivating and 4 distracting behaviors). All other children (86%) obtained a score of 2 or higher, with four children obtaining a perfect score of 5 (reporting only motivating and no distracting coping behavior).

#### Math Fluency and Calculation Competence

The average grade-equivalent score for math fluency was 4.1 (based on 23 4th-graders), and the average grade-equivalent score for calculation competence was 3.4 (based on 24 4th-graders). Thus, while students performed at grade level on fluency, they were behind on calculation competence. At the end of the program, only about half of students improved (44% for math fluency and 50% for calculation competence). Again, this finding (whether positive or negative) cannot be attributed to the tablet practice exclusively. After all, these children participated in daily math instruction during school, and thus should improve in math fluency and calculation competence, with or without the tutoring program. These post-test results are nevertheless included here to highlight the challenge of math learning for children who are already behind in math.

#### Facilitator Exit Survey

The following attributes were used to describe the strengths of the app: the app was convenient (45%), the app was exciting or fun to the students (30%), the app provided immediate feedback (25%), the tablets engaged or interested the students (15%), and the app motivated the students (5%). Facilitators also stated that students preferred practicing on the tablets compared to practicing using the worksheets. The weaknesses reported by the facilitators were that the students’ preference to work on the tablet distracted them from the worksheets (35%) and that there were problems related to technology glitches and Wi-Fi connectivity (25%).

### Setting 4: After-School Program

#### Informal Observations

Initial student buy-in to this program was a significant challenge. Throughout the first few weeks of this intervention, our team often had more facilitators than children present. Once children became more aware of the program and got to know the facilitators, more children became involved on a consistent basis. In fact, many students formed distinct bonds with the adult facilitators. However, consistent student attendance remained a challenge throughout, as this intervention was not offered within a program students were already attending. Over the course of our intervention, improvements could be observed in overall student engagement, attendance, and performance. For example, one student initially experienced extreme difficulty engaging in math practice. The student would often merely guess on problems and present little affect to the facilitators and the program in general. By the end of this program, however, the student began expressing excitement toward the app and even math practice in general. In fact, the student said to the facilitator, “Come on already, I want to practice some math!” This transformation in student behavior and attitude was a common narrative for many students, pointing to the potential benefit of IMP.

#### Systematic Observations

Student attendance in this setting was voluntary and highly variable, ranging from one to sixteen hours of participation (*M* = 6.8). Students overall felt that the sessions were fun or super fun (80%). The most commonly practiced subject was multiplication (28%), followed by fractions (23%) and addition (16%).

#### Math Attitude Survey

Many students stated that they liked at least some math (79%), while almost half of the students reported disliking at least some math (42%). About half of the students stated that they felt happy or super happy when it was time for math (53%) and about a third of the students reported that they would feel happy or super happy if they would never have to do math again (32%). Almost half of the students reported that they would like to have a job that requires a lot of math (47%), and almost half of the students thought that they were good or very good at math (53%). More students believed girls are good or very good at math (63%) compared to boys (42%). In terms of coping strategies, the average coping score was 3.36, the lowest score being 0 (one student), and only one student obtaining a score of 1 (reporting 4 positive coping strategies and three negative coping strategies). All other students obtained a 2 or higher (90%), with one student obtaining a perfect score of 5 (thus reporting 5 positive coping strategies and no negative coping strategies).

#### Math Fluency and Calculation Competence

At the onset of the program, all students performed below grade level on calculation competence (100%), and over half of them performed below grade level on math fluency (55%). Students’ scores improved at the end of the program, but more so for calculation competence than math fluency. Specifically, post assessments revealed that on average students improved more than half a grade level on calculation competence, with over half of them improving more than one grade level (55%). For fluency, only two students improved more than half a grade level.

#### Student Exit Survey

Eleven of the students felt that the program helped them either a little bit or a lot with math. The students also reported that they enjoyed staying after school to attend the program, and 89% of the students stated that they would participate in the program again. When assessing what they enjoyed about the program through open response, comments included: “I like how if I messed up they would push me to try again,” “[The program] made math fun, they made everything fun” and “[The program] taught me math and raised my grade.”

### Summary of Results

Students enjoyed the tablet-based format and often became actively engaged in solving the math problems. For example, most students reported that sessions were either “fun” or “super fun” (Settings 2 and 4). Students did not feel nervous to practice math on the tablets (Setting 3), and they explicitly commented on how much liked the program (Setting 4). Furthermore, many students believed that the intervention helped them improve their math skills (Setting 4). Increases in standardized math scores lend support for this sentiment. Teacher perceptions of the program underscore the benefit of tablet-based learning and the individualized method of practice (Setting 2). And facilitators described the tablet-based practice as convenient to implement and fun for the students (Setting 3). Challenges pertained to dealing with occasional frustrations of students and with establishing long-term practice. Finally, both teachers and facilitators indicated structural issues of Wi-Fi connectivity as a significant challenge.

## Discussion

The impetus for our study came from what we refer to as the math-practice gap: IMP is far less prevalent in discussions of academic support than informal reading practice. While reading practice is promoted through libraries and the nation-wide Accelerated Reader program, math practice is confined to a formal context of curriculum-based learning. There is also very little research on math practice, leaving many questions open, including the kind of math practice that is needed, how to promote it, who to target, for how long to carry it out, and how to interface it with other education activities. The current paper is an initial step to begin this conversation, looking specifically at the feasibility of tablet-based math practice.

As discussed in the introduction, math practice faces far more challenges than reading practice. Specifically, math is exquisitely challenging for the mind, far more than reading. The mind needs to fluidly switch between different meanings of one and the same symbols, cued by the smallest of prompts. Given such difficulty, large individual differences are likely to occur (e.g., Berch and Mazzocco, 2007), with some children needing more help than others. As a result, large-group practice becomes problematic, leading to a spiral effect of less competent students falling further behind. Yet, an individualized math program is time-consuming to prepare: an adult needs to create math problems that are tailored to the competence of each child, correct the math problems the child solved, and provide meaningful feedback (Kucian et al., 2011). It does not help that children who are already behind in math – those who need math practice most – might be least likely to enjoy math practice.

We hypothesized that tablets with math apps could be a medium by which to address the math-practice gap. Using a CBPR design, the current study is a first attempt to look at whether such tablets are feasible for low-SES elementary-school children. The CBPR approach does not allow for the precise control of variables. For this reason, several different settings were used that incorporated tablets. In each case, children were given a tablet outfitted with the math app IXL. Differences in settings pertained to the amount of time children spent with the app, the number of children present, the number of adult volunteers present, whether they had a choice for alternative activities, and whether attendance was voluntary. Results show that the app was highly engaging for children, not a single student reporting difficulty with the mechanics of using the tablet. Across all settings, whether during a one-time event or in a year-long program, virtually all students were continuously willing to practice math, often deeply engaged in math practice, and showing very few behavioral problems. When students had a choice to practice math with the tablet or on a paper-and-pencil worksheet, they preferred the tablet.

The efforts required on the part of the adults were minimal. Facilitators were largely naïve to math education, and, far from being experts in math, they often commented on their own struggles with math. The training we provided varied from a brief 2-min introduction to the program to a multi-hour mandatory training. Yet, facilitator success was similar across the board. For example, one-on-one facilitators who had the least training (Setting 1) did not report any more problems than one-on-one facilitators who had the most training (Setting 4). Even parents and family members who came in to work with their students (Setting 2) could support their student’s math practice, despite only having a brief introduction to the program and the app. Thus, the tablet-based practice was exceedingly easy for adults to supervise. At the same time, there were important caveats with the tablet-based math practice – most notably with how to promote long-term adherence which will be discussed next.

The most obvious challenge of the tablet-based practice is the cost associated with its use. This includes not only the cost for tablets and their maintenance, but also the fee for the app and the cost to maintain a reliable internet connection. For the current study, we provided a tablet and app for each student. Even so, we ran into difficulty with internet availability in all four settings. A slow internet connection led to aggravation on the part of the students, and sometimes we were required to abandon math practice on the tablets all-together. Once we left a setting, taking along the tablets, there was no alternative for the children to continue the IMP. A substantial investment in infrastructure would be needed to make tablet-based math practice a reality.

We found that the tablets and the math practice app provided reliable engagement for students to complete math problems. Thus, short-term motivation was high, once students got started. However, long-term motivation was more difficult to instill. In the after-school setting (Setting 4), where it was up to the child to attend the program, adherence issues became most obvious, with several children attending no more than three times throughout the year. Students often commented on the pressure they felt having to complete their homework, thus lacking the time to do extra math practice. Given that the tablet-based practice was not integrated with ongoing school activities, many students objected that is was not relevant to the required school work. In other words, even though the app led to a substantial amount of math practice once students started, the relation between the informal-practice progress and school work progress was not obvious to students.

Ultimately, math practice has little intrinsic motivation, other than the pleasure of one’s own progress (e.g., being able to complete a problem set). Students in our study were often quite sensitive to how far they had fallen behind and what it would take for them to reach grade-level competence. These motivational aspects stand in sharp contrast to informal reading practice, which allows students to choose a story from a vast array of stories. Even students who fall behind in reading can enjoy a story, likely to be unaware of how long it would take to reach grade-level competence. It is clear that intrinsic motivation for math practice needs to be increased, maybe by using a reward system that is similar to the Accelerated Reading Program.

Given that math practice has very little intrinsic appeal—despite the use of the app and tablets—we explored various ways to encourage adherence via reward. This included playful competition (Olympia competition during the one-time enrichment of Setting 1), snacks upon completion of a problem set (Setting 2 summer program), or prizes at the end of the program (Setting 3 after-school program). While these initiatives had some positive effects, measurable success is likely to be limited. Instead, main motivation appears to have been supplied by the adult volunteers. In fact, when the student-adult ratio was one-to-one (Settings 1, 3, and 4), math practice worked best (judging by children’s engagement). In contrast, in the settings in which there were many more children than adults (Setting 2), some behavioral problems became apparent.

Recall that adult volunteers were instructed to refrain from trying to convey math concepts to the students. This includes refraining from explaining a wrong answer and from working on the math problem for them. The facilitators’ task was instead to merely help students find an appropriately challenging problem set and motivate them to complete it (or help them adjust the challenge level, as needed). The outcome was a successful partnership where children were motivated to complete their math. Telling were our observations in the program that was carried out during school hours, when students were partnered one-on-one with adults (Setting 3): The tablet was perceived as an effective practice tool, both by the students and the tutors. It remains to be seen what it takes to improve motivation when a one-on-one facilitator setting is not possible.

Would it help to integrate informal tablet-based practice with ongoing school activities? Such integration would allow students to see their tablet practice translate into success during homework or graded assessments, rather than a mere add-on. Of course, this can be a challenge too, given that some students need more practice than others. If students would be assigned math practice that is too difficult for them, or if it would take them too long to become proficient at a concept, the positive effects of the practice are likely to fade. A better option might be to start math practice early in a child’s schooling, before large gaps appear, and instill a commitment to individualized math practice that is ongoing and independent of reaching a specific goal. Future work must determine if this recommendation holds up empirically.

## Conclusion

Our observations across four settings show that elementary-school students were highly engaged in the tablet-based math practice. This is impressive on several grounds. First, children who underperform in math might try to avoid math practice, certainly when it comes to practicing outside of formal schooling. Indeed, many of our students scored below grade level at the onset of our program, yet they often looked forward to our intervention. Second, many of the children who participated in our program reported negative attitudes toward math, something that should further increase resistance to IMP. The tablet and math app allowed them to practice math despite these attitudes. Thus, our study is a first step to demonstrate that tablets with math apps can be a feasible way to deliver sorely needed math practice, thus a way to address what we had coined as the “math-practice gap”. While our data do not speak to the relative efficacy of different aspect of the math intervention, our findings provide an important impetus for further investigating tablet-based math practice.

## Ethics Statement

This study was carried out in accordance with the recommendations of the guidelines of the Institutional Review Board, with written informed consent from all subjects. All subjects gave written informed consent in accordance with the Declaration of Helsinki. The protocol was approved by the Institutional Review Board of the University of Cincinnati.

## Author Contributions

SS designed and carried out two of the four settings outlined in the paper, outlined the first draft of the final paper, and edited as the paper was developed. MC and ZA each designed and implemented one of the four settings, contributed to the first draft of the final paper, and edited the paper as it was developed. JC was involved in the design and implementation of two of the settings, as well as the drafting and editing of the final paper. HK contributed to the design, implementation, and well as oversaw all four of the settings, assisted in the drafting of the paper, and all edits involved.

## Conflict of Interest Statement

The authors declare that the research was conducted in the absence of any commercial or financial relationships that could be construed as a potential conflict of interest.
